# Prevalence of ventilator-associated pneumonia and bacterial isolates in mechanically ventilated dogs

**DOI:** 10.3389/fvets.2025.1677280

**Published:** 2025-10-13

**Authors:** Kaitlyn Dreese, Jacob Wolf

**Affiliations:** College of Veterinary Medicine, University of Florida, Gainesville, FL, United States

**Keywords:** mechanical ventilation, ventilator associated pneumonia (VAP), dog, antimicrobials, antimicrobial resistance

## Abstract

**Introduction:**

Mechanical ventilation is used to treat respiratory failure in veterinary patients. Ventilator-associated pneumonia (VAP) is a reported complication of mechanical ventilation in both human and veterinary medicine. VAP can lead to increased length of mechanical ventilation, longer hospital stays, and increased mortality. While there are no gold-standard diagnostic criteria, the CDC has proposed surveillance guidelines for human medicine. A modified version of these guidelines has been created for veterinary medicine. The goal of our study was to determine the prevalence of VAP according to the CDC VAP surveillance guidelines, the modified veterinary guidelines, and clinician suspicion.

**Methods:**

The medical records at an academic institution were searched for patients mechanically ventilated over 24 h.

**Results:**

None of these patients met the CDC VAP surveillance guidelines or the modified guidelines for veterinary medicine. Twelve of 71 cases were concerning for possible VAP based on clinician suspicion. The most common organism grown in both the group with clinician suspected VAP and the group without was *Pseudomonas aeruginosa*, eight of which were resistant to fluoroquinolones.

**Discussion:**

It is likely that VAP is either over or under diagnosed in this population, as the clinician suspected VAP is based on subjective criteria. Findings suggest that avoiding fluoroquinolones may be beneficial when selecting an empiric antibiotic for cases in which VAP is suspected. Future studies should assess adaptions to the modified VAP surveillance guidelines for veterinary medicine because having guidelines that are too strict could eliminate cases altogether.

## Introduction

Mechanical ventilation (MV) is an important tool used for the treatment of veterinary patients with hypoxemic respiratory failure, hypercapnic respiratory failure, cardiovascular collapse, or neurologic disease (1, 2, 3, 4, 5). However, MV can lead to complications such as ventilator-associated pneumonia (VAP), acute respiratory distress syndrome, pneumothorax, and ventilator-induced lung injury (VILI) (1, 2, 4, 6, 7). VAP has been defined in both human and veterinary medicine as a new pneumonia that develops 48 h after intubation and initiation of MV (1, 3, 6, 8–16).

Development of VAP in human medicine has been associated with an increased length of hospital stay, increased duration of MV, and longer duration of ICU hospitalization (7, 8, 15–17). Additionally, appropriate antimicrobials are imperative for treatment. Therefore, accurate diagnosis is necessary for proper antimicrobial stewardship and patient care, as an increase in mortality has been shown with inappropriate antimicrobial therapy (12, 18). Human literature has reported gram-negative bacilli are often cultured on airway wash and antibiotic therapy should be tailored to cover the commonly grown microbes within that hospital’s setting (17, 19).

In human medicine, the incidence of VAP ranges between 4 and 42% (9–12). Despite multiple guidelines attempting to more accurately diagnose VAP, there is still no gold-standard criteria (12). The Center for Disease Control (CDC), the Clinical Pulmonary Infection Score (CPIS), the Johanson’s criteria, and the American College of Chest Physicians have all used varying criteria including temperature, white blood cell count, changes in ventilator settings, radiographic changes, and microbial cultures to diagnose VAP which still prove insensitive and non-specific (12).

**Figure 1:**
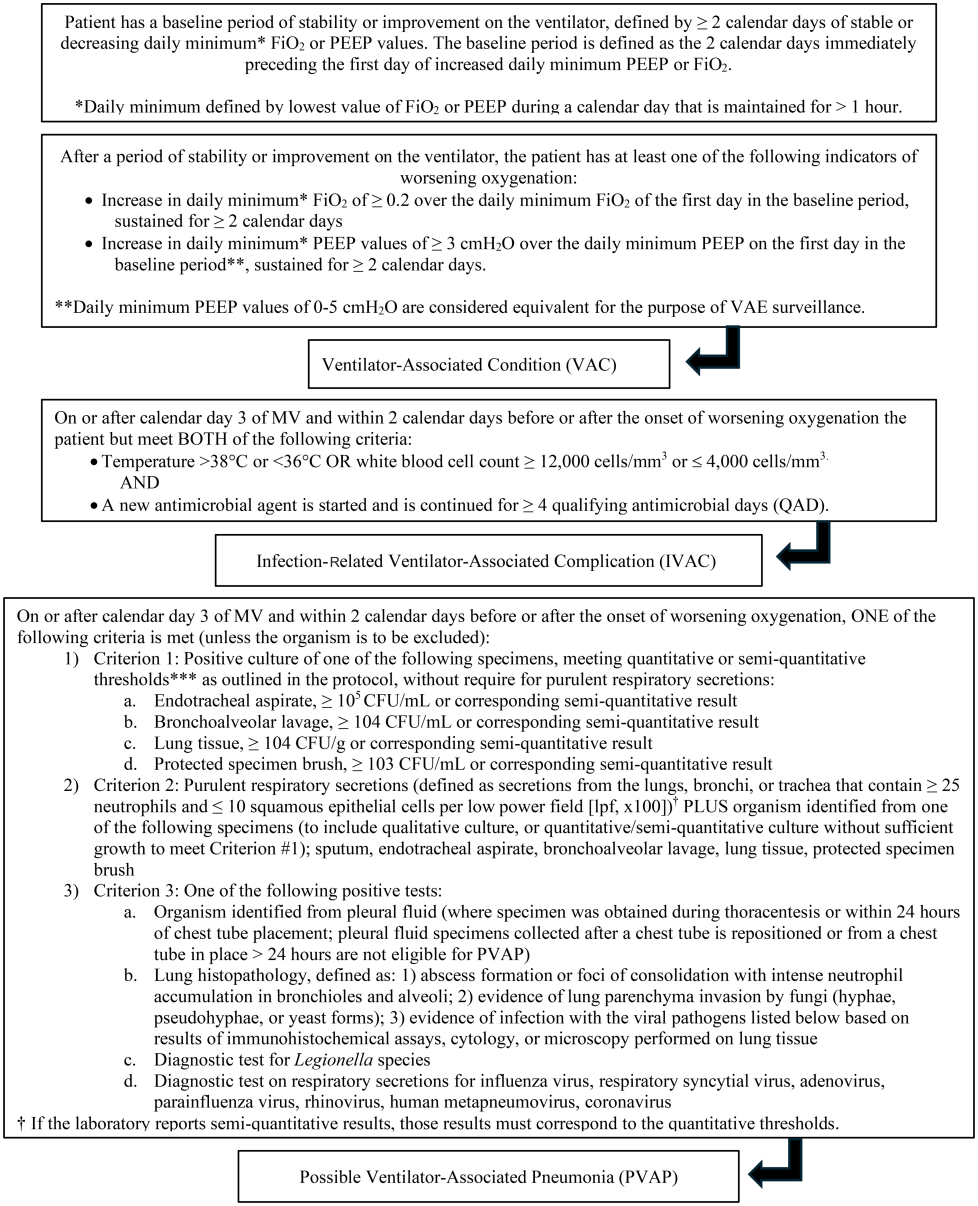
The VAP surveillance guidelines proposed by the CDC.

**Figure 2:**
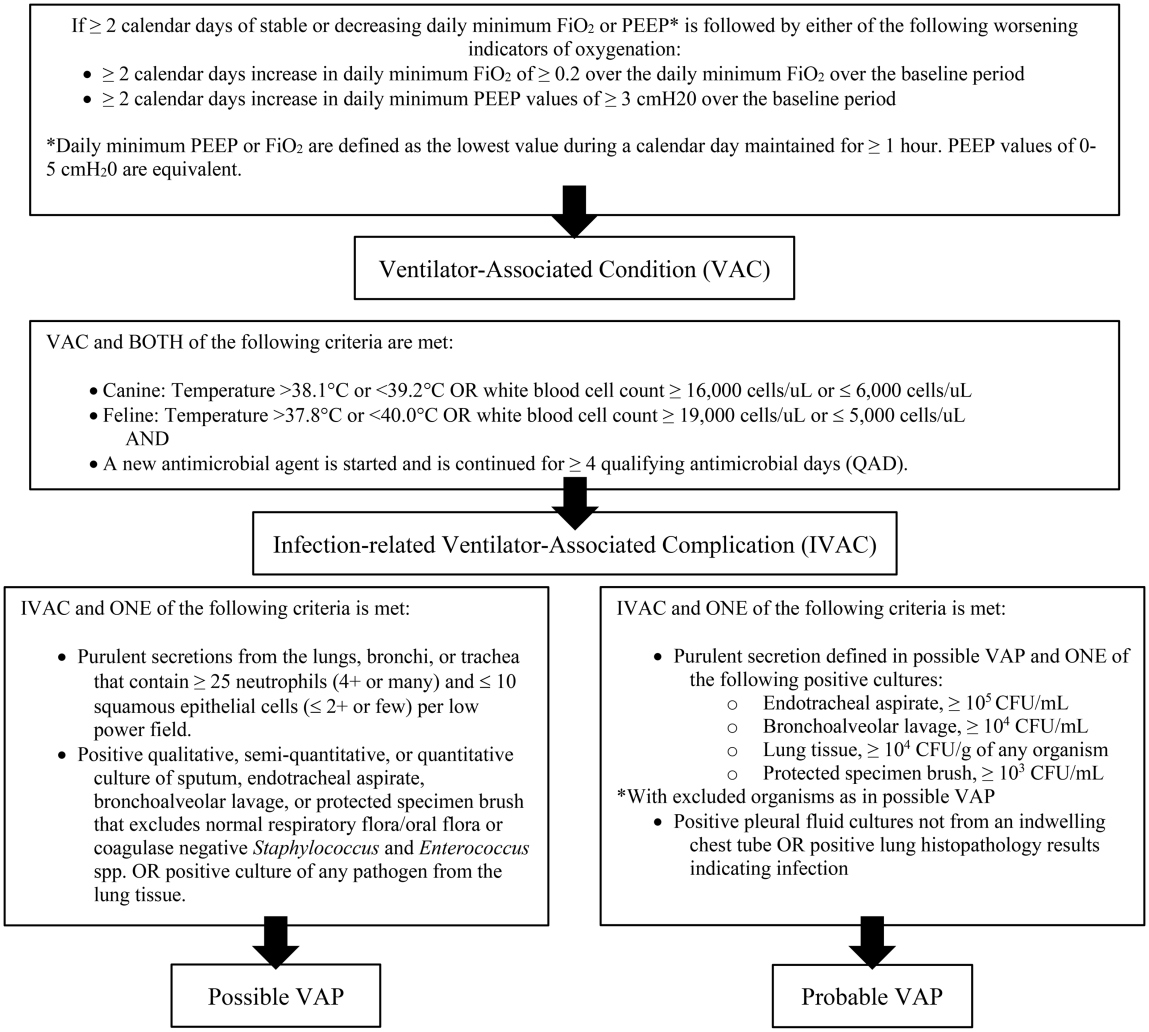
The modified VAP surveillance guidelines for veterinary medicine.

Diagnosis of VAP is challenging in both veterinary and human medicine due to the lack of objective definitions (7, 14, 20, 21). The criteria used to identify VAP are non-standardized, and different studies use different guidelines, making comparison difficult. The CDC has proposed a surveillance algorithm to identify ventilator associated events (VAE) that encompass both infectious and non-infectious events (Figure 1). The CDC defined a VAE via a combination of objective criteria: deterioration in respiratory status after a period of stability or improvement on the ventilator, evidence of infection or inflammation, and laboratory evidence of respiratory infection (7). There are three tiers of VAE: ventilator associated condition (VAC), infection-related ventilator-associated complication (IVAC), and possible VAP (PVAP) (7). These guidelines are for use in adult humans, but adaptations have been made for veterinary medicine. Modified guidelines for the diagnosis of VAP have been proposed in veterinary medicine and include a three-tiered algorithm similar to the CDC (Figure 2) (14).

There is little information about the prevalence of VAP in dogs. The objective of this study was to attempt to identify cases of VAP using the CDC surveillance guidelines, the modified guidelines for veterinary medicine, and those based on clinician suspicion, and describe the organisms found on bacterial culture.

## Materials and methods

The medical records of an academic referral hospital were searched to identify dogs that were mechanically ventilated between March 1, 2011, and January 1, 2025. Records were obtained by searching the electronic medical record for the “ventilator set-up” charge to identify dogs who were placed on the mechanical ventilator. Dogs were included if they were mechanically ventilated for over 24 h. Data collected from the records included the patients age, weight, sex, castration status, species, breed, diagnosis, initial, highest, and lowest PEEP and FiO_2_ settings, length of MV, antibiotics used, quality of secretions (none, serous, or purulent), if a fever developed [>102.5 °F (39.2 °C)], WBC count (≥ 16,000 cells/uL or ≤ 6,000 cells/uL), radiographic imaging, microbiology testing, organism growth, if there was clinical suspicion for VAP, and outcome (euthanized, died in hospital, or discharged).

Criteria based on the CDC surveillance guidelines for VAP (Figure 1) and the modified guidelines for veterinary medicine (Figure 2) were used to retrospectively to determine if VAC, IVAC, PVAP were present. Additionally, clinician suspicion was noted if there was discussion of VAP within the medical record which was based on if the dog had worsening ventilatory status [need for increased FiO_2_ ≥ 0.2 or positive end expiratory pressure (PEEP) ≥ 3 cmH_2_O], developed a fever (same criteria as above), inflammatory leukogram (same criteria as above), had pulmonary infiltrates on thoracic imaging or were culture positive. Multidrug resistance (MDR) was defined as resistance to three or more classes of antimicrobials to which the organism is usually susceptible.

## Results

There were 223 total charges for “ventilator set-up” that were identified. Of these, 17 were cats, one was a ferret, and one was a spider monkey and all were excluded. There were 19 duplicate ventilator charges for the same dog’s set-up which were also excluded. Mechanical ventilation was performed for less than 24 h in 114 dogs, and they were subsequently excluded. Seventy-one dogs met the inclusion criteria of MV for more than 24 h.

### Patient characteristics

Twenty-seven were spayed females, five were intact females, 29 were neutered males, and 10 were intact male. Twenty-three dogs were placed on the ventilator for coral snake envenomation; 11 each for cervical myelopathy and unknown diagnosis; three for collapsing trachea, ivermectin toxicity and aspiration; two for brachycephalic airway crisis, botulism, and meningitis of unknown origin, and one each of non-cardiogenic pulmonary edema, pulmonary hypertension and chronic lower airway disease, pulmonary edema of unknown origin, brain abscess, traumatic brain injury, atlantoaxial luxation, brain tumor, hepatic encephalopathy, polytrauma with pulmonary contusions and pneumothorax, intratracheal mass and hemorrhage, and bronchopneumonia.

### CDC surveillance guidelines and modified guidelines for veterinary medicine

To meet the given timeline of stable or improving on the ventilator for ≥ 2 days and then having worsening oxygenation for ≥ 2 days, dogs needed to be ventilated for at least 96 h. Only 13 of 71 cases met this requirement. Eight of the 13 cases never required an increase in PEEP or FiO_2_. Of the five cases that did require changes to their PEEP or FiO_2_, three only required an increase for a few hours and two never reached stability for ≥ 2 days. None of the cases met the criteria for VAC and subsequently IVAC, PVAP, or probable/possible VAP.

### Clinician based assessment

Of the 71 cases, 12 were considered to have clinician suspicion for VAP. Nine of these cases were not ventilated long enough to have ≥ 2 days of stability with ≥ 2 days of an increased PEEP or FiO_2_ to meet the VAC requirements. Only three of those 12 cases were ventilated long enough to be considered for a VAC. Of those cases, they were not considered VAC because one did not require increases in PEEP or FiO_2_, one case did require an increase from three cmH_2_O to eight cmH_2_O but was able to be weaned off the ventilator in less than 2 days after, and one only required an increase in PEEP on days 1–3 of ventilation and was able to be weaned.

Only four of the 12 dogs with clinician suspected VAP developed hyperthermia and 20 of 57 without clinician suspected VAP became hyperthermic. Ten of the 12 dogs with clinician suspected VAP had a CBC performed and six of the 10 had an inflammatory leukogram. Forty-six of the 59 dogs without clinician suspected VAP had a CBC performed and 28 of the 46 had an inflammatory leukogram. Eleven of the 12 cases with clinician suspected VAP had thoracic imaging performed, eight of which had concern for pulmonary infiltrates. Forty-nine of the 59 cases without clinician suspected VAP had thoracic imaging performed, 27 of which had concern for pulmonary infiltrates. This was either a new or progressive pulmonary infiltrate after starting ventilation or radiographs that were taken for the first time after initiating ventilation.

Ten of the 12 cases with clinician suspected VAP had microbiology cultures performed from an airway wash. One culture had no growth. Three cases had monomicrobial growth. Six had polymicrobial growth, three of which grew more than two types of bacteria. Three cultures grew *Pseudomonas aeruginosa* not susceptible to fluoroquinolones, and one grew *P. aeruginosa* that was susceptible to fluoroquinolones. Two cultures also grew *Escherichia coli*, one that was susceptible to fluoroquinolones and one that was not, beta-hemolytic *Streptococcus* spp., *Proteus mirabilis*, mixed flora, and *Klebsiella pneumoniae*. One of each of the following were cultured: alpha-hemolytic *Streptococcus*, *Staphylococcus pseudintermedius*, *Enterococcus faecalis*, and *Morganella morganii*.

Thirty of the 59 cases without clinical suspicion of VAP had cultures performed, seven of which had no growth. Sixteen had monomicrobial growth and ten had polymicrobial growth, two which grew three types of bacteria. Six cultures grew *P. aeruginosa*, only one of which was susceptible to fluoroquinolones, four cultures grew beta-hemolytic Streptococcus group G, three grew *E. coli*, MDR [extended spectrum beta-lactamase (ESBL), carbapenem-resistant], and mixed flora, two grew *E. coli*, *S. pseudintermedius*, and *Enterobacter cloacae*. An unknown gram-negative organism, MDR *Enterococcus durans,* MDR *E. faecalis*, *Bordetella bronchiseptica*, ESBL *E. coli*, *Pasteurella multocida*, *K. pneumoniae* susceptible to fluoroquinolones, *K. pneumoniae* not susceptible to fluoroquinolones, ESBL *K. pneumoniae*, *Enterococcus faecalis*, methicillin-resistant *S. pseudintermedius*, *S. schleiferi*, *S. pneumoniae*, and *S. angenosis* were each cultured once.

Antibiotics that were initially started, and final antibiotic prescriptions, are listed in Table 1.

**Table 1:** Organism grown from each culture with the initial antibiotics and final antibiotics started for each case.

Organism	Initial antibiotics	Final antibiotics
*E. coli* Beta hemolytic *Streptococcus*	Ampicillin/sulbactam, enrofloxacin	Ampicillin/sulbactam, enrofloxacin
Alpha hemolytic strep Beta hemolytic *Streptococcus* *S. pseudintermedius*	Ampicillin/sulbactam	Ampicillin/sulbactam, enrofloxacin
*P. mirabilis* *P. aeruginosa* *E. faecalis*	Ampicillin/sulbactam	Ceftazidime, ampicillin/sulbactam
E.coli*	Cefazolin then clindamycin and enrofloxacin	Trimethoprim/sulfamethoxazole
*K. pneumoniae* P. aeruginosa*	Ampicillin/sulbactam, enrofloxacin	Marbofloxacin
Mixed flora	Ampicillin/sulbactam	Ampicillin/sulbactam
P. aeruginosa*P. mirabilis	Ampicillin/sulbactam, enrofloxacin	Ceftazidime
Mixed flora	Ampicillin/sulbactam, enrofloxacin	Ampicillin/sulbactam, enrofloxacin
P. aeruginosa*K. pneumoniaeM. morganii	Ampicillin/sulbactam	Marbofloxacin
P. aeruginosa*	Cefazolin	Ampicillin/sulbactam
Gram negative organism	Ampicillin/sulbactam, amikacin, imipenem	Imipenem
P. aeruginosa*	Ampicillin/sulbactam	Ampicillin/sulbactam
*E. coli*	Ampicillin/sulbactam	Ampicillin/sulbactam
BordetellaBeta hemolytic *Streptococcus*	Ampicillin/sulbactam	Ampicillin/sulbactam
*E.cloaciea*Beta hemolytic *Streptococcus*	Ampicillin/sulbactam	Ampicillin/sulbactam
*E. durans* MDR	Ampicillin/sulbactam, enrofloxacin, imipenem	Imipenem
P. aeruginosa*E. coli ESBL	Ampicillin/sulbactam, enrofloxacin	Ampicillin/sulbactam, enrofloxacin
Enterobacter spp.	Ampicillin/sulbactam, enrofloxacin	Ampicillin/sulbactam, enrofloxacin
*E. coli* P. aeruginosa*	Ampicillin/sulbactam	Enrofloxacin, gentamicin spray
Beta hemolytic *Streptococcus*	Ampicillin/sulbactam	Ampicillin/sulbactam
Beta hemolytic *Streptococcus*	Enrofloxacin	Enrofloxacin
Mixed flora	Ampicillin/sulbactam, enrofloxacin	Ampicillin/sulbactam, enrofloxacin
*E. coli* ESBL, CRE	Ampicillin/sulbactam, then ceftazidime	Meropenem, trimethoprim/sulfamethoxazole
*P. aeruginosa*	Metronidazole, imipenem	Imipenem
*Pasteurella*	Ampicillin/sulbactam, enrofloxacin, ampicillin	Clindamycin, ceftazidime
Mixed flora	Ampicillin/sulbactam, doxycycline	Ampicillin/sulbactam, doxycycline
*Staphylococcus pseudintermedius* P. aeruginosa*	Enrofloxacin	Enrofloxacin
K. pneumoniae* MRSP Staphylococcus *Schleiferi*	Ampicillin/sulbactam, enrofloxacin	Ampicillin/sulbactam, enrofloxacin
*E. coli* *Streptococcus angenosis* *E. coli* *E. coli* ESBL, CRE *E. faecalis* MDR	Clindamycin, ceftazidime	Azithromycin, ceftazidime
		Chloramphenicol, metronidazole
Streptococcus *pneumoniaeStaphylococcus pseudintermedius*	Ampicillin/sulbactam, enrofloxacin	Ampicillin/sulbactam, enrofloxacin
*E. coli* *E. faecalis* *K. pneumoniae* ESBL	Ampicillin/sulbactam, imipenem	Enrofloxacin, chloramphenicol
Mixed flora	Ampicillin/sulbactam	Ampicillin/sulbactam, enrofloxacin

*Denotes the organisms that were resistant to fluoroquinolones. Those with clinician suspected VAP are in grey.

Eleven of the 12 cases (92%) with clinician suspected VAP survived and were discharged while one case was euthanized. Thirty-one of the 59 (53%) without clinician suspected VAP survived to discharge. Of those that did not survive to discharge, 23 of the 59 cases without clinician suspected VAP were euthanized and five died in hospital.

## Discussion

This study evaluated both the CDC surveillance guidelines and modified guidelines for veterinary medicine to attempt to identify cases of VAP. None of the 71 cases met the criteria for a VAC, and therefore, were not considered to have VAP under traditional definitions. This contrasts with the clinician assessment, as clinicians were suspicious for VAP in 12 of 71 cases (17%). The clinician suspicion frequency is similar to what has been reported in human medicine (9–11). Only three of the 13 cases fit the suggested timeline of stable or improving for ≥ 2 days and an increase in PEEP or FiO_2_ for ≥ 2 days to be considered VAC.

Very few studies on the incidence of VAP have been performed in veterinary medicine. However, Fox et al. reported 46% of patients met their criteria for VAP and used a newly positive culture from endotracheal lavage after 48 h on MV as diagnostic criteria (12). Another study by Cagle et al. used a modified version of the CDC surveillance guidelines without the requirement of an FiO_2_ ≥ 0.2 and PEEP ≥ 3 mmHg from baseline for ≥ 2 days (6). That study found 14% of cases fit their VAP criteria (6). A third study by Lee et al. found that 33% of cats undergoing MV over 24 h developed VAP according to their criteria which included growth on an endotracheal lavage sample or worsening alveolar pulmonary disease after initiating MV (4). Clearly, significant variability exists in the veterinary literature on the diagnosis of VAP and consistent guidelines are needed.

Diagnosis of VAP is challenging as the clinical signs are similar to other forms of pneumonia or pulmonary disease and there is a lack of objective criteria for diagnosis in both human and veterinary medicine (20, 21). In humans, the sensitivity and specificity of using clinical signs for the diagnosis of VAP has been reported as 69 and 75%, respectively (15, 22). The use of clinical signs, laboratory findings, and imaging results to diagnose VAP were inaccurate and neither sensitive nor specific when compared against lung histopathology (22). The CPIS, which combines temperature, white blood cell count, presence and quality of respiratory secretions, PaO_2_/FiO_2_ ratio, and thoracic radiographs to identify VAP, was also found to perform poorly (16, 22).

The CDC surveillance guidelines were developed to aid in identification of VAP and standardize reporting for better monitoring practices, not to use for the clinical diagnosis of VAP (20). These criteria do not always align with those used in the clinical setting to diagnose VAP and too stringent of criteria may underestimate the incidence of VAP (20). One advantage of the CDC surveillance guidelines is that it takes into consideration the patient’s ventilatory status and both infectious and non-infectious complications (22). Even still, the diagnostic criteria used to define VAP in human medicine is varied and based on a combination of clinical signs, laboratory results, and imaging (20). While none of the 71 cases were considered to have VAP based on the CDC surveillance guidelines and those modified for veterinary medicine, we attempted to identify cases suspicious for VAP based on clinician suspicion and clinical signs. Biomarkers, such as procalcitonin, C-reactive protein, IL-8, and IL-1beta, may also have a future role in the diagnosis of VAP (15, 16).

Only four of 12 (33%) cases with clinician suspected VAP and 20 of 59 (34%) cases without clinician suspected VAP had an increased temperature. Increased temperature may not be specific to VAP, and a dog may become hyperthermic while on MV due to agitation, pain, other infections (i.e., urinary tract infection) and systemic inflammation. Of the 10 cases that had a CBC performed, six (60%) of the cases with clinician suspected VAP and 28 of the 46 (61%) cases without clinician suspected VAP had an inflammatory leukogram. Again, an increased white blood cell count, may not be specific to infection and may be increased in response to both stress and systemic inflammation. Of the 11 cases that had thoracic imaging, eight (73%) of those with clinician suspected VAP and 27 of the 49 (55%) cases that had thoracic imaging without clinician suspected VAP had evidence of new or worsening pulmonary infiltrates. In human literature, thoracic radiographs have been 88.9% sensitive and 26.1% specific for VAP and may lead to misdiagnosis as other conditions like pulmonary contusions, edema, acute respiratory distress syndrome, and atelectasis can appear similarly (15).

Of the 10 dogs in which VAP was clinically suspected that had cultures performed, one (10%) had no growth while seven of the 29 (24%) cases that were cultured in the group without clinician suspected VAP had no growth. The most common bacteria cultured in the group with clinician suspected VAP was *P. aeruginosa* (19%) that was typically not susceptible to fluoroquinolones and *E. coli* (13%), beta-hemolytic *Streptococcus* (13%), *P. mirabilis* (13%) and *K. pneumoniae* (13%). This is similar to what has been reported in the literature for people with VAP in which gram-negative organisms have frequently been reported including *K. pneumoniae*, *Acinetobacter baumanii*, *P. aeruginosa*, *E. coli*, and other *Enterobacteriaceae*; gram positive organisms have also been reported including *S. aureus* and *Enterococcus* spp. (15–17, 19, 21). In a study by Maebed et al. *K. pneumoniae* (46.7%) was the most common isolate followed by *Acinetobacter* (16.7%) (17). It may be difficult to determine the pattern of growth in cases of VAP as many of the patients are already on antibiotics that cover for gram-positive coverage, affecting the culture results (17).

This contrasts with some of the reports in veterinary medicine. In several cases of VAP reported by Fox et al. *Stenotrophomonas maltophilia,* beta-hemolytic *Streptococcus, Staphylococcus aureus, Enterococcus* spp.*, Staphylococcus haemolyticus*, and a yeast species were grown on cultures (12). It is interesting to note that *Stenotrophamonas maltiphilia* has been reported in cases of VAP in humans (17). In the cases suspected to have VAP reported by Cagle et al. *Mycoplasma* spp., *Enterococcus faecium,* coagulase-negative *Staphylococcus*, and *E. coli* were grown on cultures (6). It must be noted that both studies diagnosed VAP in different ways and were performed in a different facility with a different patient population and different species and therefore may not be directly comparable. However, given the similarity of the culture results in the present study with what is found in humans with VAP, these patients may truly represent dogs with VAP. This should prompt concern that the modified CDC guidelines may underrepresent VAP diagnoses in dogs.

Bacteria cultured in the group that was not suspected to have VAP included *E. coli* (20%) with one being and ESBL and two an ESBL/CRE, *P. aeruginosa* (17%), only one of which was susceptible to fluoroquinolones, and beta-hemolytic *Streptococcus* (Group G) (11%). The mixed flora in these cases likely represented contamination. There were three additional drug resistance microorganisms including an MDR *Enterococcus*, ESBL *K. pneumoniae*, and a methicillin resistant *S. pseudintermedius*. A study by Tso et al. described microorganisms cultured from dogs that were mechanically ventilated due to tick paralysis and found that *E. faecalis, S. pseudintermedius, E. coli, K. pneumoniae, E. faecium, P. aeruginosa, Enterobacter cloacae* and *Mycoplasma* spp. were cultured most frequently (5). *Staphylococcus* spp., *Streptococcus* spp., *Klebsiella* spp., *A. baumannii, A. hydrophila, S. rubidaea, P. mirabilis, Pasteurella* spp., and *Candida* were cultured only once (5). Only one of the 26 animals was classified as having VAP after a positive culture 4 days after intubation with radiographic evidence of pneumonia; the remainder were classified as aspiration pneumonia. Patients undergoing MV may have an increased risk of aspiration making differentiating worsening respiratory disease versus VAP difficult.

In a study that evaluated isolates from dogs with aspiration pneumonia, the most common isolates were *E. coli* (38%), *Mycoplasma* (21%), *Pasteurella* (19%), *Staphylococcus* sp. (17%), *Streptococcus* spp. (12.8%), *Klebsiella* spp. (12.8%), *Enterococcus* spp. (10.6%), *Bacteroides* spp. (6.4%), *Fusobacterium* spp. (4.3%), *P. aeruginosa* (4.3%), and single cultures with growth of *Corynebacterium* sp., *Hemophilius* sp. *Peptostreptococcus* sp., and *Serratia marcescens* (23). Another study that assessed culture growth in suspected aspiration pneumonia found growth of *E. coli, K. pneumonia, P. multocida, S. canis, K. oxytoca, Acinetobacter* spp.*, Neisseria weaver*, and *Frederikenia canicola* (24). In the present study, many of the bacteria cultured from the group without clinician suspicion of VAP were similar to what has been found in those with VAP. In both groups, there was a large percentage that grew *P. aeruginosa*, which has not been commonly reported in other veterinary literature. Ideally, larger studies would be performed that compare the difference in bacterial growth between dogs with VAP, dogs with hospital acquired pneumonia, and dogs with community acquired pneumonia.

Selection of appropriate antibiotics when VAP is suspected is vital for rapid and effective treatment. Inappropriate antibiotic choices are a common cause of treatment failure in VAP that leads to an increased risk of mortality (15, 22). In human medicine, *P. aeruginosa* is one of the most common bacteria associated with VAP with a prevalence of about 4% (18). VAP with MDR *P. aeruginosa* has been identified as a predictor of hospital death and initial inadequate antibiotic therapy (18). It has also been reported that 70% of *E. coli* and *K. pneumoniae* produce ESBL and 20% of the *E. coli* and *K. pneuomniae* found in VAP patients in the United States have extended spectrum cephalosporin resistance (17).

In humans, it is recommended to start treatment that includes coverage for *S. aureus*, *P. aeruginosa*, and other gram-negative bacilli (16). The risk of giving antibiotics when no infection is present and delaying antibiotic treatment when infection is present needs to be weighed for each patient. The challenges in diagnosing VAP make this a pressing issue as up to 67% of patients may be treated for VAP that do not actually have VAP (22). Severity of clinical signs, rather than just on the basis of independent signs like fever, leukocytosis, radiographic changes, and culture results, should inform the decision to start empiric antibiotics (22). A more conservative approach may be taken for patients who are hemodynamically and respiratory stable.

Of the cases that were suspected to have VAP, only three (33%) of the nine did not have their antibiotics changed that had cultures performed that grew microorganisms. Three (33%) cases underwent escalation therapy and two (22%) had antibiotics changed based on culture results. One was changed from enrofloxacin to marbofloxacin a day before discharge for an unknown reason. Of the 20 cases that were not suspicious for VAP that had cultures performed and grew microorganisms, 13 (65%) did not undergo antibiotic changes. Five (25%) of 20 cases underwent escalation therapy and two (10%) of the cases underwent changes in antibiotics based on culture results. In a study by Fox et al., 69.2% of patients underwent antimicrobial changes based on culture results on an endotracheal wash (12).

There is a growing concern for antimicrobial resistance, especially in patients who have been in hospital for a long period of time or on antibiotics previously. In human medicine there are several risk factors that increase the risk of MDR VAP including use of IV antibiotics within the last 90 days, five or more days of hospitalization prior to VAP, septic shock at the time of VAP, and renal replacement therapy prior to VAP (16, 23). In the current study of dogs suspected to have VAP, three of the cultures grew a *P. aeruginosa* not susceptible to fluoroquinolones and one grew an *E. coli* not susceptible to fluoroquinolones. For the cultures not suspected to have VAP, five cultures grew *P. aeruginosa* that was not susceptible to fluoroquinolones, two grew an MDR *Enterococcus* and *E. coli* ESBL/CRE, and one culture each grew *K. pneumoniae* not susceptible to fluoroquinolones, an ESBL *Klebsiella,* ESBL *E. coli*, and MRSP.

While, antimicrobial resistance patterns are usually hospital dependent, it may be reasonable to avoid empiric prescription of fluoroquinolones for gram-negative coverage for dogs undergoing MV with suspected VAP. Four of the 9 patients with clinician suspected VAP that had culture performed had grown gram negative bacteria resistant to fluoroquinolones, 3 of which were started enrofloxacin and later changed to a different antibiotic.

There are several limitations to this study. Culture results and resistance patterns are likely to be specific to specific hospitals and may not be extrapolated to other locations. Additionally, the retrospective nature makes it difficult to determine why diagnostics were performed or treatments were changed. While ventilator settings are usually modified to maintain the SpO_2_ 90–97%, PaO_2_ between 60–100 mmHg, and PCO_2_ 35–50 mmHg, it is not always apparent for the exact reasoning settings were changed in individual patients’ record. The clinician suspected VAP criteria were subjective and inconsistently considered clinical signs of the patient, deterioration while on MV, and the clinician’s interpretation. This could have over or underestimated the occurrence of VAP in the study population. The ability to accurately diagnose VAP in this manner is severely limited. Not every case had radiographs performed prior to initiating ventilation and therefore, it is difficult to determine if the animals had pulmonary infiltrates present prior to ventilation and if they were worsening. Additionally, how cultures samples were obtained was not clear within the patient record and could lead to misinterpretation of results. While samples are usually obtained via endotracheal wash after sterile placement of an endotracheal tube, cultures could have been obtained via bronchoalveolar lavage or transtracheal wash and could affect culture results. Future studies are needed in both human and veterinary medicine to identify a way to more accurately diagnose VAP in a clinical setting.

## Conclusion

The aim of this study was to attempt to identify cases of VAP using the CDC surveillance guidelines, those modified for veterinary medicine, and those with clinician suspicion of VAP. No cases of VAP were identified using the CDC guidelines or those modified for veterinary medicine and 17% of cases were concerning for VAP based on clinician suspicion. Many of the cultures grew organisms that were resistant to fluoroquinolones which should be considered in future cases when attempting to cover for gram-negative organisms when VAP is a concern. Future studies should focus on a more accurate way to identify VAP in a clinical setting and appropriate empiric antibiotic coverage; however, this may be dependent on the hospital antibiogram.

## Data Availability

The raw data supporting the conclusions of this article will be made available by the authors, without undue reservation.
